# Laser cleaning and Raman analysis of the contamination on the optical window of a rubidium vapor cell

**DOI:** 10.1038/s41598-022-19645-z

**Published:** 2022-09-15

**Authors:** Patrik Gádoros, Aladár Czitrovszky, Attila Nagy, Roman Holomb, László Kocsányi, Miklós Veres

**Affiliations:** 1 Department of Atomic Physics, Institute of Physics, Budapest University of Technology and Economics, Műegyetem Rkp. 3, 1111 Budapest, Hungary; 2 Institute for Solid State Physics and Optics, Wigner Research Centre for Physics, Konkoly Thege M. Str. 29-33, 1121 Budapest, Hungary; 3 Uzhhorod National University, Zankoveckoyi, 89a Str., Uzhhorod, 88015 Ukraine

**Keywords:** Engineering, Materials science, Physics

## Abstract

In this work, we present the laser cleaning of a Rubidium vapor cell and the Raman analysis of the contaminant material to be removed. The optical window of the vapor cell had gradually lost transparency due to the development of an opaque layer of unknown composition at the inner side during the normal operation of the cell. Laser cleaning was successfully performed by a frequency-doubled Nd:YAG laser focusing the beam inside the cell, avoiding any possible damage to the window. A single laser pulse was enough to clear away the black discoloration at the focal spot and locally restore the transparency of the window. The Raman spectra of the deposit showed peaks not yet described in the literature. Comparison with known Rubidium germanate spectra and simulation results strongly suggested that the unknown material was Rubidium silicate.

## Introduction

Rubidium vapor cells have many applications both for commercial and research purposes. The most widespread among them is the rubidium frequency standard (RFS). Based on the hyperfine split of the ground state of rubidium a crystal oscillator can be locked to 6.8347 GHz. The stability of the commercial devices is as good as 0.5–1 × 10^11^ 1/s^1^. RFS has found its applications since the 1950’s in atomic clocks, aerospace, telecommunications and aerospace industry^2,3^.

Optical magnetometers belong to the most sensitive tools to measure magnetic field. They utilize the interaction between light and a resonant medium, that is many times vapor of alkali atoms (among others rubidium) confined in a glass cell. Thanks to high accuracy and ease of use, optical magnetometers are now used in earth and material science, magnetic resonance imaging (MRI) and various other fields^4^.

Rubidium vapor is also prone to develop transverse optical patterns due to resonant light propagation. Some of these pattern types exhibit complicated dynamic behaviour such as bistability and hysteresis in switching from one to another. This phenomenon may be exploited as optical memory^5^. While nearly resonant interaction is suitable of reshaping ultrashort laser pulses^6^.

Recent results of applying rubidium plasma for laser wake field acceleration (LWFA) are encouraging. LWFA is based on the concept that a high-density plasma is created by an ultrashort laser pulse, which is followed by a wake field wave. In this wave strong electric fields are generated, which can exceed by multiple orders of magnitude the electric field of conventional particle accelerators limited by material breakdown. Therefore extremely high acceleration gradient can be achieved offering the prospect of new, compact and cost-efficient particle accelerators for research and medical applications^7–10^. Such systems, however, require high quality optical cells containing the rubidium vapor and having clear optical windows with minimal losses, as well as solutions securing long term operation of these windows by the elimination of contaminations, if any.

Laser cleaning is, in fact, the purposeful utilization of laser radiation to remove any unwanted surface layer from a substrate. By properly setting the irradiation parameters like power, wavelength, focal position and pulse duration, very precise removal of the surface layer can be attained without damage to the base material. Especially good results can be expected when the optical properties of the two materials (layer and substrate), such as absorbance of the laser radiation, differ significantly^11–13^.

Since the pioneering work of Asmus et al. in the 1970s^14^, laser cleaning has become a well-established method in the preservation of cultural heritage. Nowadays it’s widely used in restoration of historical buildings^11,13,15–19^, sculptures^20,21^ and metal artifacts^12,22^. It has many advantages to the traditional mechanical and chemical cleaning techniques, such as reduced cost, the demand for manual work and impact on the environment^13,19^.

Its efficiency, low cycle time and material effort coupled with a high level of automation made laser cleaning a lucrative alternative for industrial actors as well. Most commonly, it is used for material preparation before manufacturing in the semiconductor and microelectronic industry, machine industry and waste recycling^23–28^. In addition, there are also studies about utilization in the nuclear industry for decontamination purposes^29^.

For obvious reasons laser cleaning is most common for very rugged base materials like stone, usually limestone, granite or marble^11,13,17–19,30–34^ and metal, usually iron, steel, copper, bronze or even noble metals^12,22,23,29,34–45^. Examples of applications to sensitive objects exist but are far less frequent. This group ranges from frescos^46,47^ to samples as vulnerable as historical paper^42,48,49^ and fabric artifacts^41^ or a fusion diagnostic mirror^50^. Applications on glass substrates have also been reported^51–55^. There is also a wide range of surface layers to be removed according to the base material and purpose of the cleaning. Black crusts and graffiti are common issues for restoration of historical buildings^11,12,15,18,20,56,57^, tarnish for metal artefacts^22,41^, oxide and paint layers and contamination for material preparation^23,36,39,40,58^.

Process control of the laser cleaning is an essential factor in finding the appropriate irradiation parameters and achieving an efficient layer removal. For historical buildings, visual inspection of the cleaned surface is usually sufficient, but for more demanding samples, a complex approach is necessarily composed of optical microscopy and an appropriate selection of material analysis techniques like Raman spectroscopy, optical reflection spectroscopy, scanning electron microscopy, energy-dispersive X-ray analysis^11,16,22,37–41,44,45,49,59,60^. Laser-induced breakdown spectroscopy is a promising method to control the ablation during the cleaning process or analyze the surface before it^17,31–33,52,58,61,62^.

Contamination of optical windows of vapor cells (and other optical parts) is a well-known problem originating from these components' operational environment and conditions. The contamination has a negative effect on their optical performance since it can decrease the transmitted laser intensity, modify the wavefront of the laser pulses, and even facilitate laser-induced damage during the operation of the system by creating localized absorption. Most of such contamination consists of particulate material. Earlier studies dealing with the particle contamination of surfaces of optical elements and focusing on identifying the origin of contamination^63^ showed that contamination could originate from various sources, including particles from the surrounding environment and products of laser-induced damage of the optics itself or adjacent parts, and therefore can involve various types of materials^64^. Depending on the type of the contamination particle, its interaction with a laser pulse can be characterized by three main effects: particle removal, secondary contamination of the surface by fragments of the original contamination particle, and laser-induced damage of the host surface. The first type of interaction is, in fact, laser cleaning. This effect was used to remove particles from aluminium mirror surfaces with high efficiency by using a pulsed UV laser source^65^. However, there is no example in the literature for removing the rubidium-based layer from a glass substrate, especially not from the inner walls of a closed rubidium cell.

In this work, we present the laser cleaning procedure of a worn rubidium vapor cell together with the Raman analysis of the black discoloration. The cell was used to generate and characterize plasma in rubidium vapors by intense femtosecond laser pulses.

## Experimental details

### Sample and measurement points

**Figure 1:**
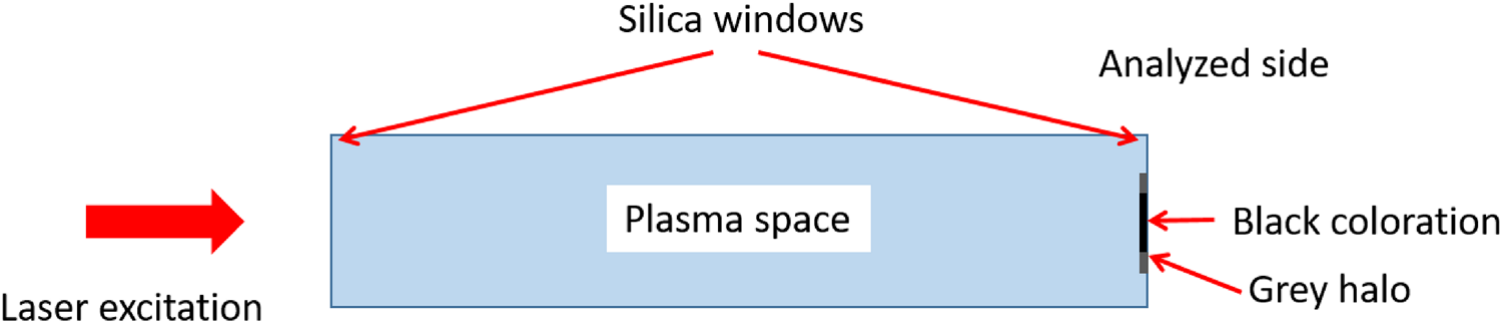
Sketch of the Rubidium cell. The direction of excitation in the homogenous plasma experiments and the cleaning laser pulses are the same. The coloration was observed on the inner side of the opposing window.

The sample was a worn Rubidium cell from a laser-induced plasma generation experiment, which was no longer in use due to the poor transparency of the exit window (Fig. 1). For the plasma generation, the glass cell filled with rubidium vapor was placed in a vacuum chamber incorporating a temperature-controlled oven. The cell is a cylindrical glass tube of 2.5 cm outer diameter with optical quality quartz end windows. The temperature of the cell and, consequently, the density of the rubidium vapor were varied during the experiments. The plasma was generated by an ionizing Ti–sapphire laser (Legend, Coherent Inc.) operating at 800 nm wavelength and 1 kHz repetition rate, with a pulse duration of about 40 fs and maximum pulse energy of 4 mJ. The laser beam was aligned longitudinally along the glass cell inside the vacuum system, and the transmitted fraction of the laser light was blocked by an appropriate beam dump^66^.

**Figure 2:**
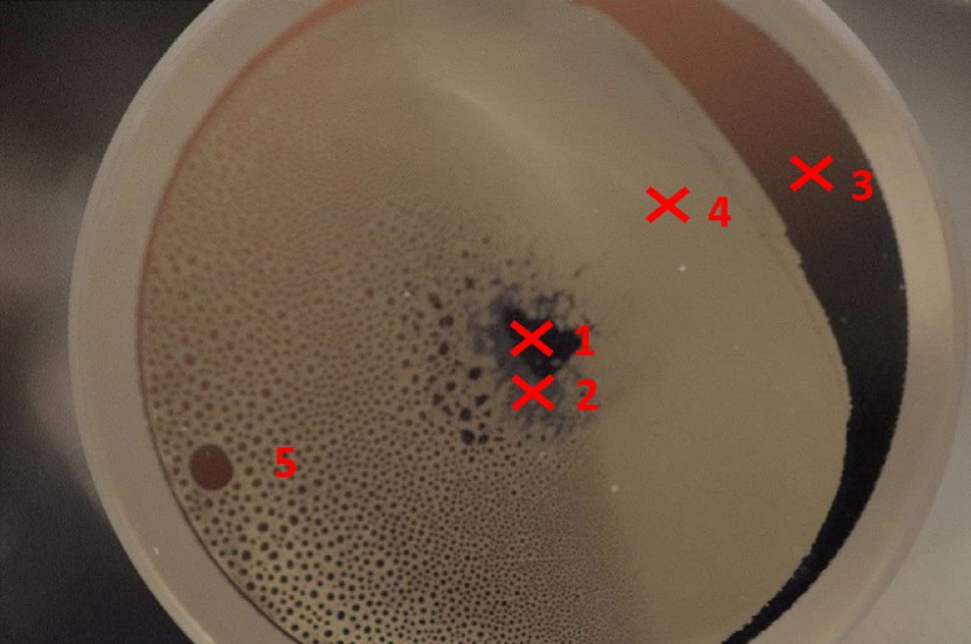
Regions and measurement points on the window of the Rubidium cell: black discolored surface (1), grey discolored surface (2), condensed rubidium as a contiguous layer (3) and droplets (5), clear silica surface (4). The analysis was performed at points 1–4 (marked by X).

The optical windows of the rubidium vapor cell are made of quartz. While these windows of a new cell are obviously clear, two types of opaque areas can be distinguished on the internal surface of the exit window of the examined worn cell (Fig. 2). The metallic and reddish color areas are of metallic rubidium deposits on the window, which are present in a continuous layer (on the right perimeter of the window, mark no. 3 in Fig. 2) and small droplets (left part, mark no. 5 in Fig. 2). It does not compromise in any form the functionality of the cell since, under operating conditions, the rubidium is in vapor phase and not present on the surface. Furthermore, in the central part of the window, an amorphous discolouration can be observed, which consists of a matte black region (mark no. 1 in Fig. 2) with a grey halo (mark no. 2 in Fig. 2). The exact composition of this layer is unknown. However, it is a plausible supposition that during the experiments, the laser pulses heated/ablated the quartz material and the emitted material interacted with rubidium forming some kind of rubidium silicates, especially if we consider the fact that the laser irradiation of the quartz window was the most intense at the area where this surface alteration occurred. Last but not least, a large intact quartz surface is present (mark no. 4 in Fig. 2), which offers an ideal location for reference measurements.

### Laser cleaning

The laser cleaning tests of the colored surface were performed by a Quantel Brilliant Q-switched Nd:YAG laser operating at its fundamental wavelength (1064 nm). The pulse width was 3.2 ns (FWHM). Various pulse energies were used during the experiments, cautiously increasing the energy from 50 to 360 mJ (maximum output of the laser in question) by setting the time delay between the flash lamp and Q-switch. The laser beam profile was Gaussian with a beam diameter of 5 mm.

**Figure 3:**
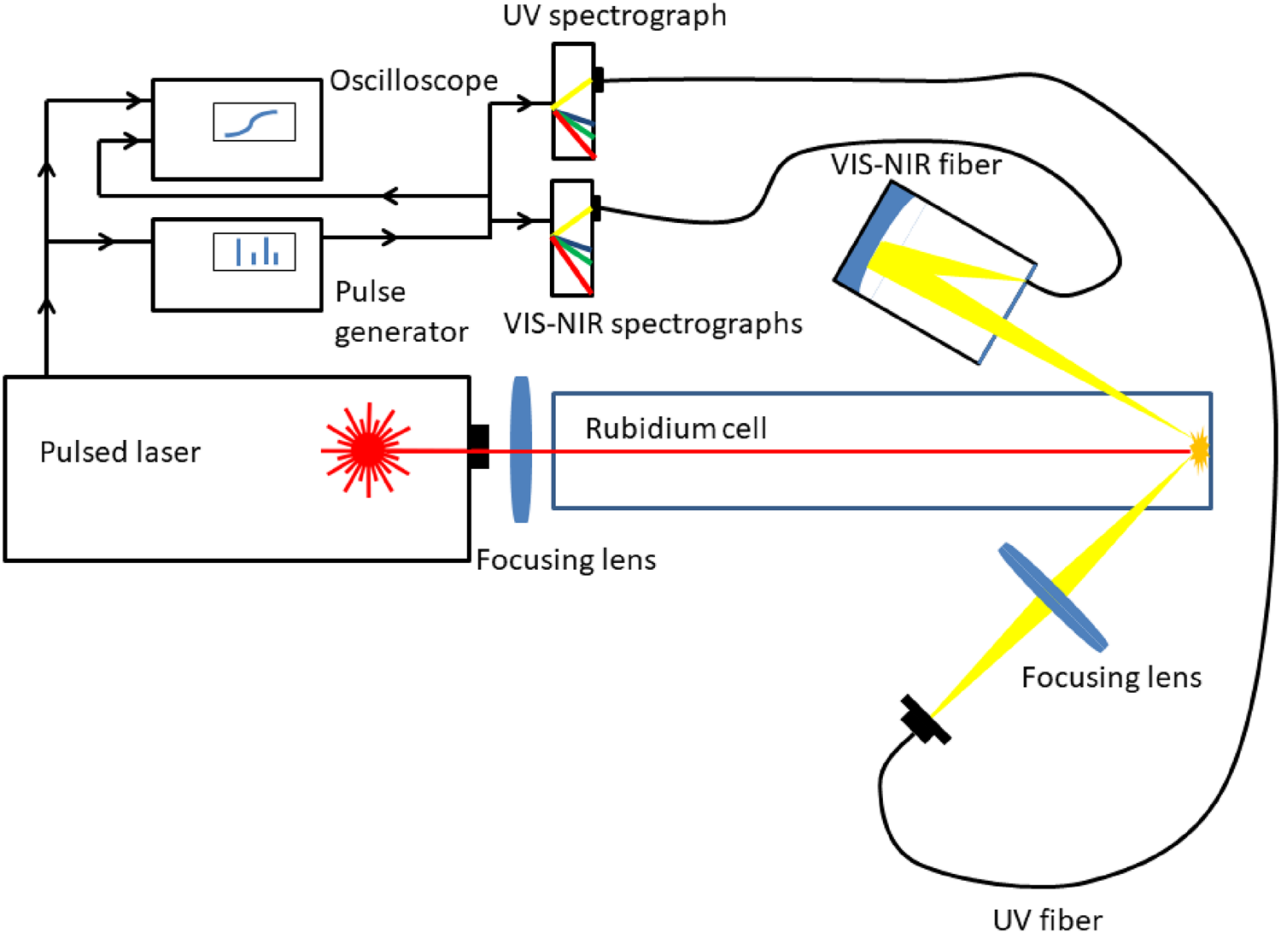
Setup of the laser cleaning of the Rubidium cell. The apparatus to capture the emission signal of microplasma eventually ignited during the removal of the residues is detailed in the figure.

The laser radiation was passing through the intact window of the cell, focused by a biconvex converging lens of 295 mm focal length to a point 1 mm in front of the contaminated surface (at the inner side of the window) to minimize heat stress to the glass material and prevent the formation of micro-cracks threatening with the destruction of the sample (Fig. 3). The laser was operated in single pulse mode for the same reason.

The fluence can be calculated to 400 J/cm^2^ (1.25 × 10^11^ W/cm^2^), assuming ideal focusing conditions and considering the 50 mJ pulse energy, 5 mm beam diameter, 295 mm focal length with 1 mm defocusing. With 360 mJ (maximum pulse energy), the fluence is around 3 kJ/cm^2^ (9 × 10^11^ W/cm^2^). These values are generally enough to ignite weak microplasmas, thus triggering shockwaves to boost cleaning efficiency and possibly enable LIBS analysis.

The irradiated surface was placed perpendicular to the laser beam. Scanning the sample in 2D was performed by a manual stage, keeping the defocusing constant and discharging only one pulse at one point of the sample.

A Stellarnet Bluewave compact Czerny–Turner spectrographs were coupled to the laser to perform LIBS analysis for enhanced control of the cleaning process if the laser irradiation generated microplasma (Fig. 3).

### Raman analysis

Raman spectroscopic measurements were performed on a Horiba Jobin Yvon portable Micro-Raman spectrometer with 532 nm excitation. The excitation laser beam was focused onto different areas of the internal surface of the optical window from the outside by using a Nikon 50× objective lens (NA = 0.75). The Raman spectra were recorded in the 200–1000 cm^−1^ region with 6 cm^−1^ resolution.

The self-consistent density functional theory (DFT) field method using the hybrid B3LYP functional consisting of a linear combination of the pure corrected exchange functional by Becke^67^ and the three-parameter gradient-corrected correlation functional by Lee et al*.*^68^ was applied for geometry optimizations of these clusters and Raman spectra calculations using the Gaussian-09 program package^69^. The Los Alamos National Laboratory (LANL) double zeta (DZ) valence basis set (LANL2DZ) was used for all atoms in the cluster models^70^. The vibrational contribution of saturating hydrogen atoms in the calculated Raman spectra of Rb_2_Si_2_O_8_H_6_, Si_3_O_9_H_6_ and Rb_2_Si_3_O_10_H_6_ clusters were eliminated as described elsewhere^71,72^. The Lorentz-shape function with the intensity proportional to the calculated Raman activity and with full width at half-height (FWHH) of 10 cm^−1^ was used to simulate the Raman spectra of clusters.

## Results and discussion

### Removal of the residues

**Figure 4:**
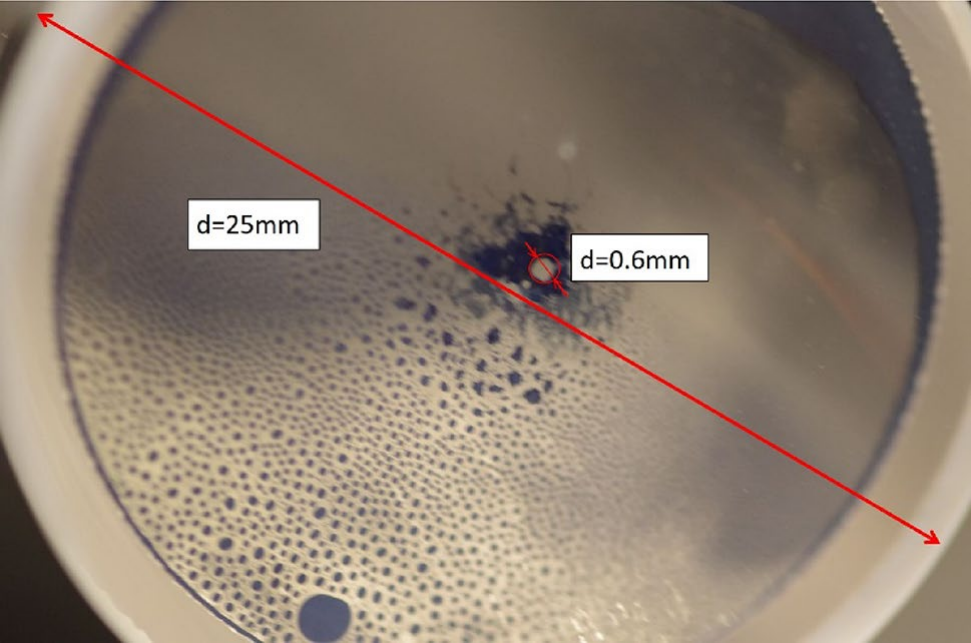
Exit window of the Rubidium cell after first cleaning shot. The black residue has disappeared around the focus point in a nearly regular circular area of 0.6 mm diameter.

The minimum laser pulse energy was applied, targeting the black surface at point 1 in Fig. 2. It was enough to restitute the window's transparency in a spot of 0.6 mm diameter (Fig. 4).

Observing no damage to the window, the fluence was gradually increased from the minimum value to the maximum (roughly 3 kJ/cm^2^, as discussed above). During the laser irradiation, the coloration of the window disappeared, which became transparent anew. The cleaned and the intact surfaces showed no difference at optical microscope inspection. During the process, no microplasma generation was observed.

Considering that the window is practically fully transparent to the laser radiation after the ablation of the surface contamination, the absorbed energy density is probably much less. It explains why no plasma was formed by experimental parameters usually suitable for LIBS measurements.

### Composition of the contamination

**Figure 5:**
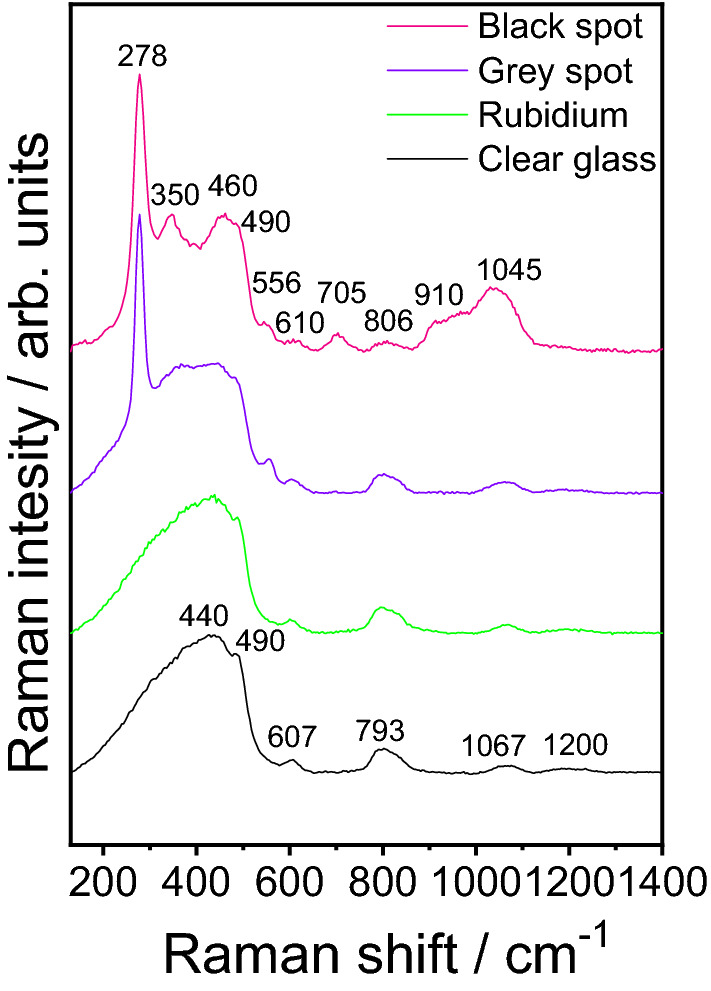
Raman spectra were recorded in different spots of the contaminated quartz window of the rubidium vapor cell.

Raman spectroscopic measurements were performed on the black central and grey outer part of the contamination, the rubidium layer and the clean glass (marked with X signs in Fig. 2) to determine the origin of the contamination in the central part of the optical window. Several Raman spectra were recorded in each area that was found to be characteristic of all four spots. These spectra are compared in Fig. 5. The spectrum of the clear glass consists of broad peaks with a high-intensity asymmetric band in the 200–600 cm^−1^ region. The Raman peaks observable in this spectrum are characteristic of fused silica. They can be attributed to the vibrations of oxygen atoms within the silica tetrahedra (at 440 cm^−1^), breathing vibrations of oxygen atoms in 4-membered rings (at 490 cm^−1^) and 3-membered rings (610 cm^−1^), respectively, various bending and stretching motions (at 793 cm^−1^) and stretching involving SiO_4_ tetrahedral units and/or Si–O–Si bonds (1067 cm^−1^ and 1200 cm^−1^)^73,74^. The spectrum recorded in the area of the rubidium film is very similar to the previous one, indicating that the metal covering the quartz window in these places does not incorporate into the glass matrix.

**Figure 6:**
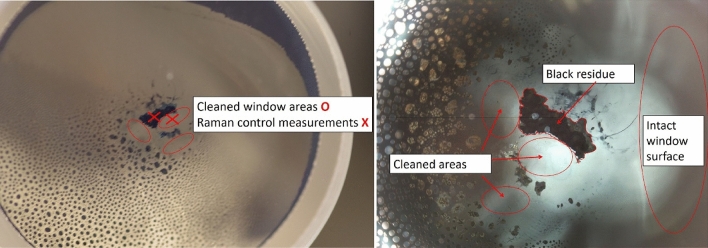
Rubidium cell window after laser cleaning; a small part of the black deposit is left for the purpose of control measurements. The Raman spectroscopy analysis was performed at the locations marked with X.

On the other hand, the Raman spectrum of the contamination is remarkably different (grey and black spots in Fig. 6). A strong and narrow peak dominates the curves of both the grey and black regions at 278 cm^−1^. Additional new bands of smaller intensity can also be detected at 350, 460, 556, 910 and 1045 cm^−1^. From these new peaks, the 350, 910 and 1045 cm^−1^ ones are present in the spectrum of the black spot only. The characteristic Raman features of the fused silica can also be observed, serving as a background for the new peaks. However, the shape of some bands differs from that observed in the clean spot spectrum, indicating the glass's structural transformation in the contamination area.

The contaminant was formed under high-energy pulsed laser radiation in a closed cell filled with rubidium vapor. Therefore, it can be assumed that the grey and black discolored residues consist of the material inside the cell, namely quartz and/or rubidium—moreover, the easy contamination removal by a cca. 3 kJ/cm^2^ nanosecond pulsed laser indicates that it is a particulate material aggregated on the inner surface of the quartz window (and not the result of the incorporation of the metal into the glass), and the new Raman peaks arise from this structure.

The effect of alkali cations on Raman spectra and the structure of SiO_2_ glasses were investigated earlier^75^. The differences in the silicate networks of (M_2_O)_x_(SiO_2_)_100-x_ glasses (M = Li, Na, K, Rb, Cs and x = 5, 10, 15, 20, 25, 30) were established and characteristic vibrational modes in the Raman spectra of alkali-silicate glasses were also reported. In general, it was found that the bands in the 900–1200 cm^−1^ spectral region result from highly localized Si-nonbridging oxygen stretching modes, allowing to determine the alkali distributions around SiO_4_ tetrahedra.

The Raman intensity of the low frequency spectral bands at 500 and 600 cm^−1^ in the Raman spectra of rubidium-silicate glasses are found to increase with increasing of Rb content. First band was assigned to highly delocalized vibrational mode of both bridging and non-bridging oxygens in the silicate network, while the second band can originate from a vibrational mode localized on a defect structure^75^. The highly localized vibrational mode at 950 cm^−l^ in the Raman spectra of rubidium disilicate glass result from Si–O stretching in SiO_4_ tetrahedral units containing two non-bridging oxygens. The spectral features at 1100 and 1150 cm^−1^ in the Raman spectra of rubidium-silicate glasses indicate the presence of two distinct structural environments in which an SiO_4_ tetrahedron contains one non-bridging oxygen^75^.

The features observed in the Raman spectrum of the contaminant can also be compared with the Raman spectra of the xRb_2_O(1 − x)GeO_2_ rubidium germanate system^74^, since the structure of silicate (quartz) glasses is similar to that of germanates (both are composed of tetrahedral—silicate and germanate, respectively). There, three peaks were reported at 250, 370 and 512 cm^−1^, the intensity of which increases with Rb_2_O content. They were attributed to the Raman active bending modes of Q^2^, and Q^1^ tetrahedra of the germanate, which become localized as the number of non-bridging oxygen atoms increases and the germanate tetrahedron starts acquiring molecular-like character. The appearance of these new peaks was also accompanied by a redistribution of Raman intensities in the 720–870 cm^−1^ region, where a broad band can be attributed to Ge–O^−^ vibrations of different Q^0^–Q^3^ germanate tetrahedrons. The shift of the Raman bands towards lower wavenumbers in this region was attributed to the decrease of Q^3^ and Q^2^ type tetrahedrons and the increase of the Q^1^ and Q^0^ species with rubidium content^74^. All these structural transformations were attributed to the incorporation of the rubidium oxide into the glass network.

Features similar to those described for xRb_2_O(1 − x)GeO_2_ can be observed in the Raman spectra recorded in different areas of the quartz window of the worn vapor cell. A comparison of the Raman spectrum of the contamination with that of the clear glass in our vapor cell shows the appearance of the 278, 350 and 545 cm^−1^ bands, accompanied by changes in the 900–1100 cm^−1^ region belonging to different Q^0^–Q^3^ species of the silicate tetrahedra. Although the peak positions are different from those observed in rubidium–germanate (mainly because of the presence of heavier Ge in the latter instead of Si, shifting the Raman peaks to lower wavenumbers), the similar changes in the Raman spectra imply the incorporation of rubidium into the silicate structure causing the increase of the amount of non-bridging oxygen and formation of molecular-like Q^2^ and Q^1^ tetrahedra. So the black contaminant is a silicate glass containing rubidium, presumably in oxide form.

**Figure 7:**
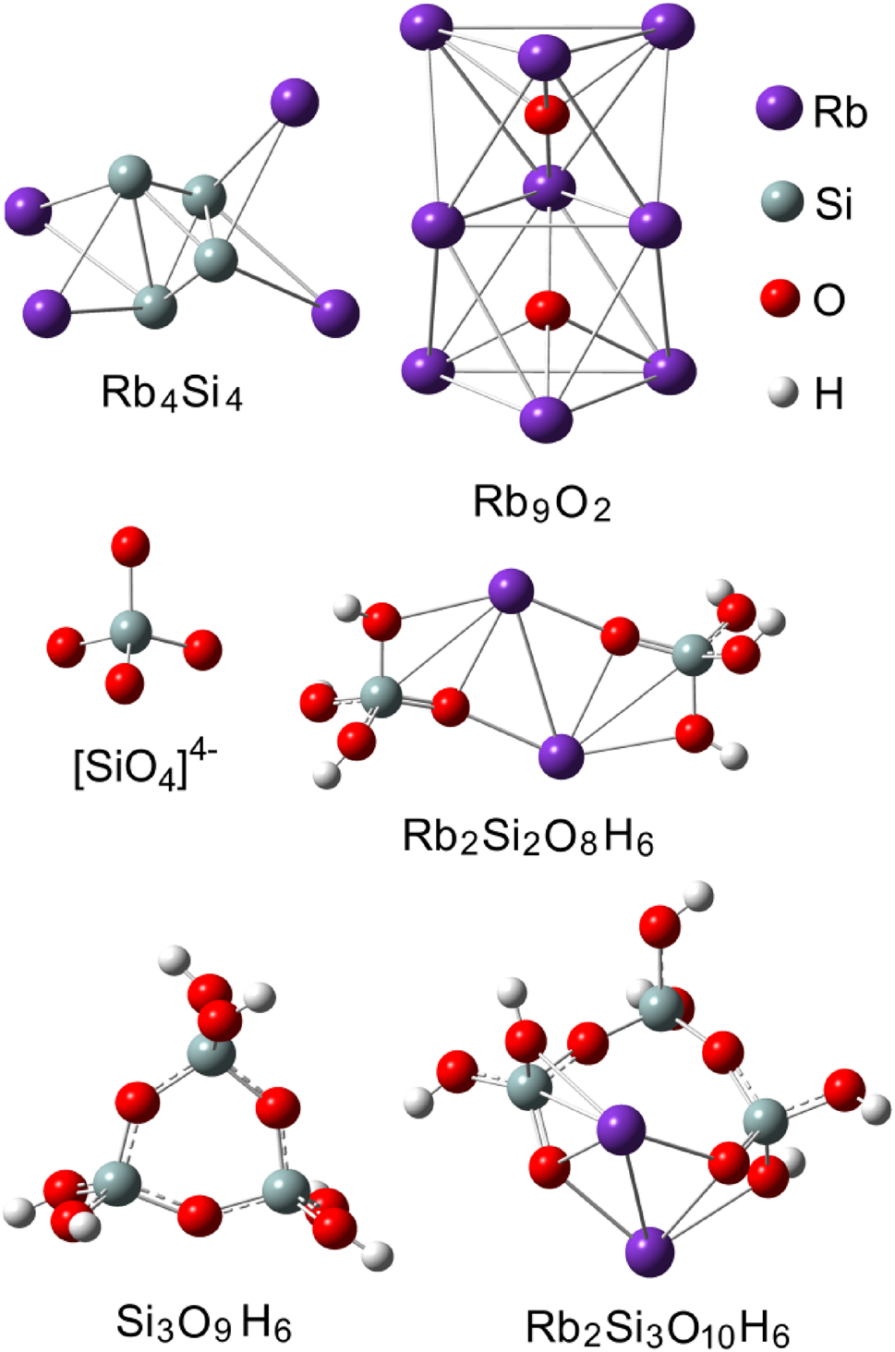
Optimized geometry structures of different Si–O, Rb–Si, Rb–O and Rb–Si–O clusters.

In order to verify this interpretation of the Raman features, the molecular modeling of different gas-phase silicon-oxide, rubidium-silicon, rubidium-oxide and rubidium-silicon-oxide clusters was performed (Fig. 7).

**Figure 8:**
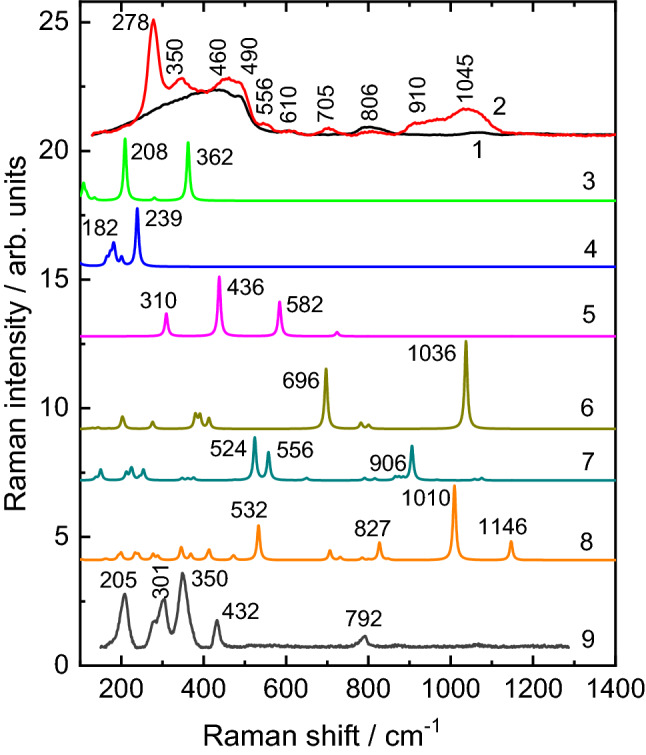
Experimental Raman spectra of rubidium cell measured in the regions of clean SiO_2_ glass (1) and contaminated black spot (2) together with the simulated Raman spectra of Rb_4_Si_4_ (3), Rb_2_O_9_ (4), SiO_4_ anion (5), Rb_2_Si_2_O_8_H_6_ (6), Si_3_O_9_H_6_ (7), Rb_2_Si_3_O_10_H_6_ (8) clusters calculated at B3LYP/LANL2DZ level of theory. The experimental Raman spectra of tridymite polymorph of silica (9) is also shown for comparison^76^.

Figure 8 show the comparison of experimental Raman spectra of rubidium cell measured in the regions of clean SiO_2_ glass (curve 1) and contaminated black spot (curve 2) together with the simulated Raman spectra of different clusters (curves 3–8). The experimental Raman spectra of tridymite polymorph of silica is also shown for comparison (curve 9). As can be seen, the vibrations of Rb_4_Si_4_ and Rb_2_O_9_ clusters can contribute to the low frequency region of experimental Raman spectra of black spot. Two Raman active modes at 208 and 362 cm^−1^ were calculated for Rb_4_Si_4_ cluster. The main characteristic Raman mode of rubidium suboxide (Rb_2_O_9_ cluster) is located at 239 cm^−1^ (Fig. 8, curves 3 and 4). Therefore, the two bands in the experimental Raman spectra at 278 and 360 cm^−1^ can be related with the presence of structures based on Rb–Si and Rb–O clusters. However, the Rb_4_Si_4_ and Rb_2_O_9_ clusters have no high frequency vibrational modes observed in the experimental Raman spectra.

The high frequency Raman modes can be related with the entire transformations of silica network surface induced by Rb ions. Therefore, the additional cluster models were used to verify this idea. First, the isolated SiO_4_ anion was used and the Raman active modes of this cluster were calculated at 310, 436 and 582 cm^−1^ (Fig. 8, curve 5). Then, the two SiO4 tetrahedra each with one non-bridging oxygen were used to model the Rb incorporation in the glass structure (cluster Rb_2_Si_2_O_8_H_6_). As can be seen, the calculated Raman modes of this cluster at 696 and 1036 cm^−1^ are in good accordance with the experimental bands at 705 and 1045 cm^−1^ observed in the Raman spectra of black spot (Fig. 8, curves 2 and 6). In addition, the model built from three corner sharing SiO_4_ tetrahedra were used to study the influence of Rb atoms on the structure and vibrational properties of simplest 6-membered ring. The calculated Raman modes of Si_3_O_9_H_6_ cluster at 524/556 and 906 cm^−1^ agree well with the experimental bands at 556 and 910 cm^−1^ observed in the Raman spectra of black spot (Fig. 8, curves 2 and 7). The presence of non-bridging oxygens (O^−^ charged defects) in the ring model can lead to the incorporation of Rb cation (cluster Rb_2_Si_3_O_10_H_6_). The main Raman active modes of this cluster calculated at 532 and 1010 cm^−1^ are in good agreement with the experimental bands at 556 and 1045 cm^−1^ (Fig. 8, curves 2 and 8). Therefore, it is possible to conclude that high frequency Raman modes at 910 and 1045 cm^−1^ in the Raman spectra of black spot can originate from Rb incorporation and subsequent network transformation (defect SiO_2_ and Rb–SiO_2_ ring formation) occurred in the near surface layers of SiO_2_ glass. Furthermore, the low frequency bands at 205, 301, 350 cm^−1^ observed in the Raman spectra of tridymite polymorph of SiO_2_^76^ (Fig. 8, curve 9) can be an additional support of the transformation of SiO_2_ network surface at presence of rubidium cation.

## Conclusion

It has been demonstrated that laser cleaning is an efficient tool for removing the contamination of the Rubidium vapor cells. The transparency of the optical window contaminated on the internal surface inside the closed cell was successfully restored with a single shot of a frequency-doubled Nd:YAG laser without inflicting any damage to the window itself. The Raman analysis of the contaminant displayed peaks, which had not been described in the literature before. Comparison of the Raman spectra of the sample with reference spectra together with calculated Raman spectra of model structures showed that the material of the discoloration consisted of Rubidium silicates formed upon irradiation of the rubidium vapor with high-intensity femtosecond laser pulses.

## Data Availability

The datasets used and/or analyzed during the current study are available from the corresponding author on reasonable request.
